# Bioinspired liquid-infused surface for biomedical and biosensing applications

**DOI:** 10.3389/fbioe.2022.1032640

**Published:** 2022-09-29

**Authors:** Yuemeng Yang, Qinglin Zhu, Li-Ping Xu, Xueji Zhang

**Affiliations:** ^1^ Beijing Key Laboratory for Bioengineering and Sensing Technology, School of Chemistry and Biological Engineering, University of Science and Technology Beijing, Beijing, China; ^2^ School of Biomedical Engineering, Health Science Center, Shenzhen University, Shenzhen, China

**Keywords:** liquid-infused surface, bioengineering, medical devices, biosensing, anti-bioadhesion, liquid-repellency, self-healing

## Abstract

Nature always inspires us to develop advanced materials for diverse applications. The liquid-infused surface (LIS) inspired by Nepenthes pitcher plants has aroused broad interest in fabricating anti-biofouling materials over the past decade. The infused liquid layer on the solid substrate repels immiscible fluids and displays ultralow adhesion to various biomolecules. Due to these fascinating features, bioinspired LIS has been applied in biomedical-related fields. Here, we review the recent progress of LIS in bioengineering, medical devices, and biosensing, and highlight how the infused liquid layer affects the performance of medical materials. The prospects for the future trend of LIS are also presented.

## 1 Introduction

Through evolution and natural selection, many organisms can accommodate the complex living environment by developing specific wettability surfaces that have unique functions in reproduction and predation (Liu et al., 2017). For instance, benefiting from lubricating water on the peristome with highly wettable microstructure, Nepenthes pitcher plants can provide the precarious foothold, thus forcing insects to aquaplane into its pitcher-like stomach (Bohn and Federle, 2004). By mimicking the wetting phenomenon of Nepenthes pitcher plant surface, tremendous efforts have been devoted to developing the liquid-infused surface (LIS) (Wong et al., 2011). Bioinspired LIS is a promising dynamic liquid surface fabricated by infusing the barrier liquid (lubricant) into various structured substrates (Wong et al., 2011; Liu et al., 2013; Cui et al., 2015; Shi et al., 2016). Once the surface energy of substrate and barrier liquid are matched, the infused liquid can be confined on the surface through capillary and van der Waals forces, creating a stable and homogenous liquid interface (Maji et al., 2020).

**FIGURE 1:**
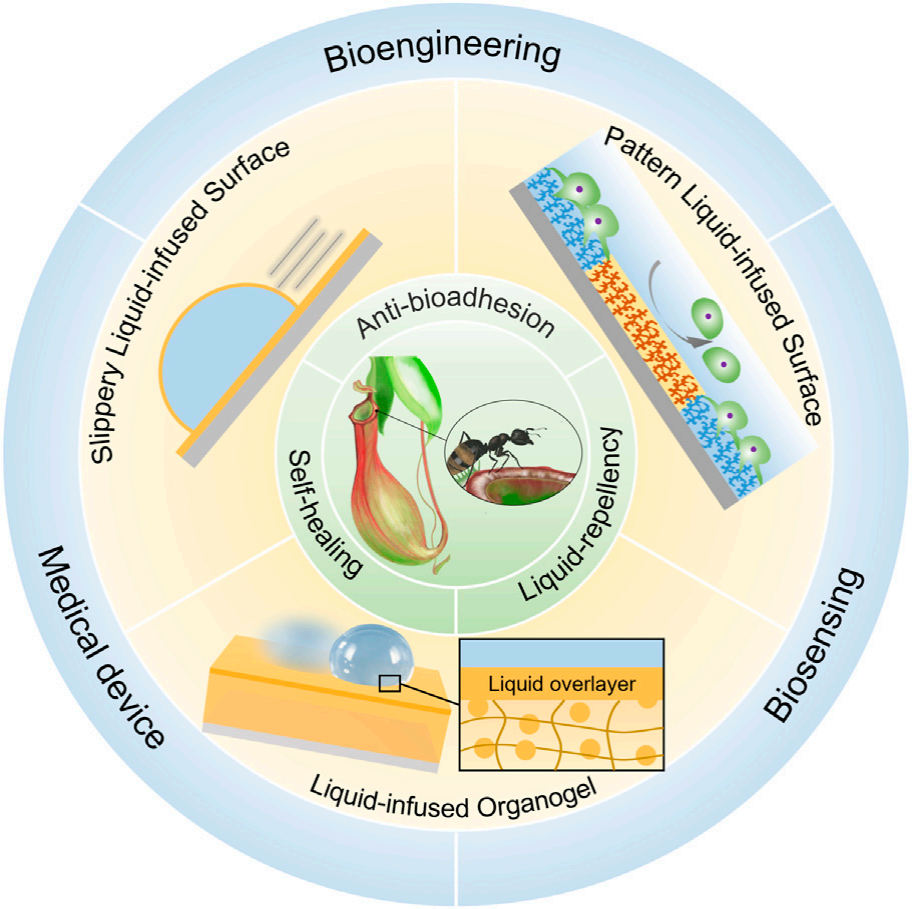
The recent progress of liquid-infused surface (LIS) inspired by Nepenthes pitcher plants. Due to their intrinsic advantages, including liquid-repellency, anti-bioadhesion, and self-healing properties, several functional LIS materials have been applied in bioengineering, medical device, and biosensing.

According to the characteristics of solid substrate, LIS can be classified into slippery liquid-infused porous surface (SLIPS), liquid-infused organogel surface (LIOS) and patterned liquid-infused surface (PLIS) (Figure 1). SLIPS proposed by Aizenberg’s group creates a molecularly slippery and omniphobic liquid interface by infusing a low surface energy liquid into the porous substrate (Wong et al., 2011). Due to the stable yet dynamic liquid interface, SLIPS can repel most immiscible liquids, even physiological fluids. And SLIPS has exhibited stable anti-adhesion capability towards nucleic acids, proteins, bacteria, cells, and marine organisms, and can function even at high pressure (Glavan et al., 2014; Amini et al., 2017; Kovalenko et al., 2017). Another type of LIS is liquid-infused organogel surface (LIOS) (Salbaum et al., 2021). The barrier liquid swells the organogel matrix through diffusion and forms a thin liquid layer that can be replenished from the liquid-storage compartments of organogel bulk (Cui et al., 2015). Compared with SLIPS, LIOS exhibits better self-healing ability due to the self-replenishment and the intrinsic properties of organogel. Besides SLIPS and LIOS, to endow LIS with new functions, patterned liquid-infused surface (PLIS) was also developed by combining the slippery liquid-infused surface with other wetting states on the same surface. On PLIS, selective regional adhesion of biomolecules can be achieved (Shi et al., 2016; Lei et al., 2020).

No matter which solid substrates were used, the main advantages include stable liquid-repellency, excellent anti-bioadhesion and self-healing ability (Figure 1). The distinctive interface is critical to these properties of LIS. The defect-free and molecularly slippery liquid interface eliminates defects that could become nucleation sites for undesired bio-adhesion and lead to pinning liquid. Furthermore, the fluidity of the infused liquid endows the LIS with self-healing, resulting in the long-term stability of various properties of the LIS. These features make LIS an emerging function material with great potential in the healthcare-related fields, such as medical tubing or implants, surgical devices, cell culture, and biomarker detection (Wu et al., 2019; Wang et al., 2022; Yuan et al., 2022). This mini-review summarized the development of LIS with its emerging applications in healthcare-related fields, including bioengineering, medical devices, and biosensing (Figure 1). Due to the length limitations, we mainly focus on how the unique liquid interface of LIS inspires and influences the development of new medical materials rather than a comprehensive review of this wide field. Finally, the challenges and prospects of LIS in biomedical and biosensing applications are also presented.

## 2 Bioinspired LIS for bioengineering

Understanding the interaction mechanisms between biological systems and artificial materials is crucial to the development of bioengineering. Benefiting from the slippery liquid interface, LIS displays unique effects on manipulating interfacial behaviors of organisms, such as adhesion, growth, and migration (Mackie et al., 2019). Hence, LIS is promising to unravel the complexity of biological systems and open up new possibilities in bioengineering applications. This section presents recent studies of LIS as a laboratory platform for regulating biofilm formation, cell culture, and tissue engineering.

### 2.1 LIS for regulating biofilm formation

Planktonic bacteria always tend to adhere and proliferate on the interface, forming sessile biofilm, which makes eradicating bacterial infections more challenging (Dunne, 2002). Recently, LIS has been shown to significantly reduce surface biofilm coverage of various bacteria under dynamic and static culture conditions (Zhu Y. et al., 2022).

The low biofilm coverage can be ascribed to the inhibited early adhesion of bacteria on the liquid interface because firm adhesion is the first critical step in biofilm formation (Dunne, 2002). During exposure to the external environment, through maintenance of infused liquid layer at the material surface, LIS restricts the direct contact of bacteria with the material and provides a weakly adhesive interface for bacteria. (Epstein et al., 2012; Doll et al., 2019). Therefore, the integrity of infused liquid layer is crucial for the anti-biofilm properties of LIS. Doll et al. evaluated the correlation between LIS anti-adhesion performance and liquid layer stability by screening four different structures and five different lubricants (Doll et al., 2017). The effect of different LIS on biofilm coverage can be observed by confocal laser scanning microscopy (CLSM). Only the spike-structure surface with maximum roughness combined with lubricants of intermediate viscosity could maintain a stable and intact liquid layer underwater, resulting the minimal biofilm coverage. In addition, although LIS was covered with biofilm in a static culture of *Botryococcus braunii*, the biofilm did not firmly adhere to the liquid interface and can be removed easily (Howell et al., 2014).

Although LIS shows an excellent anti-biofilm adhesion effect against various bacteria, the biofilm formation process is still complex and species-dependent (Kovalenko et al., 2017). It was reported that drug-resistant *Pseudomonas aeruginosa* exhibited higher biofilm coverage on LIS (Li et al., 2013). Recently, Levkin’s group has employed the PLIS for bacterial culture to investigate the mechanisms of biofilm formation at the liquid interface (Bruchmann et al., 2017; Lei et al., 2019; Lei et al., 2020). On PLIS, bacteria did not only grow on isolated hydrophilic spots but spread over the liquid-infused periphery. Biofilm bridges connecting adjacent biofilm microclusters can be observed using fluorescence microscopy. Based on this observation, Lei et al. further studied the bacterial spreading and biofilm formation process of a wide variety of bacteria on PLIS. It was demonstrated that extracellular DNA and nutrients are essential for bacteria to overcome repulsion and biofilm formation on the liquid interface (Lei et al., 2019).

Due to the significant role of LIS in the formation process of bacterial biofilms, LIS provides a valuable platform for revealing structure–function relationships in biofilms and studying interactions of biofilms with various medically relevant materials.

### 2.2 LIS for cells and tissues regulation

Similar to bacterial adhesion, the LIS can reduce cell adhesion on materials by maintaining a stable barrier liquid layer. It was demonstrated that LIS could prevent or reduce the diverse types of cells adhering despite varying cell properties (Yao et al., 2014; Schlaich et al., 2018; Yong et al., 2018). In addition, a small number of cells settled on the LIS have a rounder morphology that can be removed by weak shear forces, indicating that cells do not firmly adhere to LIS and remain resting (Ueda and Levkin, 2013; Leslie et al., 2014; Yuan et al., 2015). It is worth noting that LIS is not cytotoxic and has no effect on external cell viability by examining macrophage viability, phagocytosis, and bactericidal activity (Chen et al., 2017). Based on these features, LIS has displayed potential for spatially and temporally controlling the behaviors of cells.

**FIGURE 2:**
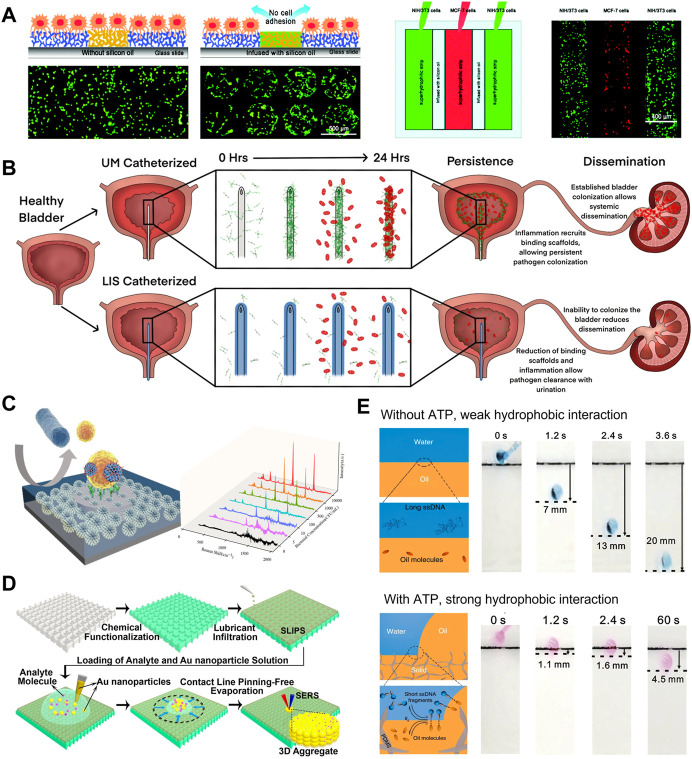
**(A)** The cell repellency of silicone-oil-infused barriers is superior to air-assisted superhydrophobic surfaces. PLIS can be used to culture different types of cells on the same substrate. (Shi et al., 2016). Copyright 2016, Wiley-VCH **(B)** In the mouse model of urinary tract infection, liquid-infused silicone-catheter decreased bacterial colonization on the catheter surface and within the bladder, enabling effective intervention for urinary tract infections (Andersen et al., 2022). Copyright 2022, eLife Sciences Publications Ltd **(C)** Patterned liquid-infused nanocoating that combined the hydrophobic anti-bioadhesion periphery and hydrophilic sensing regions can be used for sensitive bacterial SERS detection (Wang et al., 2022). Copyright 2022, American Chemical Society **(D)** Due to the liquid-repellency, analytes can be enriched on LIS by evaporation of droplets, enhancing subsequent SERS signal (Yang et al., 2016). Copyright 2016, The Authors, Published by the National Academy of Sciences **(E)** The sliding angle of RCA droplet on LIS can be controlled by the chain length of ssDNA. The sliding behavior of the droplet has been used as the output signal to detect ATP (Gao et al., 2020). Copyright 2020, Nature Publishing Group.

PLIS which combines the different functional domains on the same surface has been used to create cell microarrays. Our group fabricated PLIS on superhydrophilic–superhydrophobic patterned substrate by infusing silicone oil on the hydrophobic part (Shi et al., 2016). Stable NIH/3T3 cell arrays formed even after incubating under water for 12 h, which demonstrated that the silicone oil-infused barrier exhibited the long-term cell repellency (Figure 2A). While under the same culture condition, cell migration could be observed on superhydrophobic regions due to the poor stability of the air-assisted superhydrophobic barrier. Moreover, the co-culture of multiple cells can be realized on PLIS, since silicone oil-infused barrier can effectively avoid cells cross-contamination.

In addition to repelling individual cells, LIS is effective in preventing the adhesion of cell tissues (Zhang et al., 2018). The strategy used for cell sheet tissue engineering *via* LIS has been revealed (Juthani et al., 2016). The mesenchymal stem cells can grow and proliferate to form cell sheets on LIS by depositing the fibronectin layer. In the presence of excess silicone oil on LIS, the cell sheets can be easily detached due to the negligible adhesion of the cell sheet to the underlying substrate.

LIS has been validated as a controllable medium for manipulating organism behaviors at the liquid-liquid interface. In addition, by further careful design, this system also could emerge as a promising laboratory tool to study cell-to-cell interactions, tissue engineering, cell propagation, drug evaluation and other applications.

## 3 Bioinspired LIS for medical devices

Biological fouling on medical device surfaces has become a severe and persistent issue, which often causes device failure and severe clinical complications. However, most materials with anti-biofouling properties cannot meet the requirements of long-time use in complex environments, which can be ascribed to the decomposition or damage of functional molecules that buttress anti-adhesion properties (Banerjee et al., 2011; Falde et al., 2016; Erathodiyil et al., 2020).

Recently, bioinspired LIS has been investigated and used in medical devices to resist the fouling of undesired proteins, cells, platelets, and pathogens. As discussed in Section 2, biofouling cannot attach to LIS and can be subject to passive removal. Compared with other solid antifouling surfaces, LIS demonstrates its superiority in long-term stability. Epstein et al. presented that LIS prevents diverse biofilm accumulation over 1 week or longer under low flow conditions, which is 35 times greater than the best-case scenario for PEG surfaces (Epstein et al., 2012). And similar effects were also observed in the culture of HeLa cells (Ueda and Levkin, 2013). Long-term anti-adhesion properties of LIS can be ascribed to the self-healing of its liquid interface. Therefore, various attempts have been made to fabricate medical devices with LIS coating to relieve clinical complications caused by biofouling.

Various biomedical implants have been developed to replace a missing biological structure or support a damaged biological structure, together with multiple functions including medical monitoring and drug delivery. However, biofouling on implants in the complex human body environment leads to the failure of implants and many complications. Thus, the fabrication of biomedical devices with the stable anti-biofouling coating is urgent. Leslie et al. designed a PVC medical tubing in which the interior is immobilized with a liquid layer, which could remains patency for 8 h without heparin (anticoagulant), similar to other heparin-based coatings (Leslie et al., 2014). In another study, liquid-infused silicone-catheters reduces uropathogens colonization on the its surface by disrupting fibrinogen deposition (Andersen et al., 2022). As shown in Figure 2B, in the mouse model, LIS inhibited the colonization within bladder and systemic dissemination of uropathogens, enabling effective intervention for urinary tract infections. In addition to the remarkable results of medical tubing, the subcutaneous implants with infused liquid coating demonstrate outstanding potential *in vivo*. Upon implantation in rats, SLIPS-modified implant limits bacterial infection and vastly reduces local inflammation (Chen et al., 2017). Furthermore, the LIS has been integrated into surgical and diagnostic devices. The scalpel blades with LIS coating showed lower blood and *E. coli* adhesions after simple washing, thus avoiding cross-contamination (Tesler et al., 2015). Constructing LIS coatings electrosurgical instruments significantly reduce the adhesion of soft tissues, leading to a much smaller charring wound (Zhang et al., 2018).

In recent years, by integrating the functional molecules on the liquid-infused surface, LIS has been endowed with the ability to intervene in outside environment actively (Manna et al., 2016; Badv et al., 2019b; Wang et al., 2021). Didar’s group reported a liquid-infused vascular graft with built-in bio-functional nanoprobes that promote implant endothelialization without compromising the repellency properties of LIS (Badv et al., 2019a). In addition, LIS-coated implants combined with controlled release of anti-inflammatory or antimicrobial drugs have also been developed to increase the longevity and safety of devices (Kratochvil et al., 2016; Douglass et al., 2021).

In general, materials with LIS coating are promising candidates to enhance the anti-biofouling properties of medical devices, which reduce the risk of inflammation and infection in clinical treatment, and avoid complications caused by the systemic administration of drugs. Moreover, LIS also exhibits excellent material compatibility. It can be directly combined with many medical-grade material surfaces permitting the retention of specific material properties such as strength or transparency. Despite the promising results of LIS *in vivo*, the cytotoxicity and the detrimental downstream effects of LIS should be thoroughly evaluated in long-term practice.

## 4 Bioinspired LIS for emerging biosensing applications

As the crucial part of biosensors, the biosensing interface strongly influences the analytical performance of biosensors (Xu et al., 2019; Zhu Q. et al., 2022). Constructing an optimal interface to achieve sensitive detection and ultratrace analysis is one of the challenges in biosensing fabrication. Recently, leveraging the unique liquid interface, LIS has exhibited immense potential as a promising biosensing interface in detecting biomarkers in complex samples.

Undesirable nonspecific adhesion is one of the obstacles preventing the application of biosensors in the detection of real samples. The emergence of patterned liquid-infused surface (PLIS) provides an ideal approach to enhance the specific recognition of targets. Yousefi et al. used the patterned biofunctional liquid-infused surface to eliminate the adhesion of interfering substances in milk at the target recognition interface, thereby lowing the detection limits of *E. coli* by 4-fold (Yousefi et al., 2021). For more complex interference systems that accompany continuous blood clotting, LIS can effectively prevent clot formation and adhesion, directly detecting interleukin six in non-anticoagulated whole blood (Shakeri et al., 2020). PLIS was also used to enhance the identification efficiency of probes and sample enrichment. Our group developed a patterned liquid-infused nanocoating that combined the hydrophobic anti-bioadhesion periphery and hydrophilic sensing regions to achieve *Staphylococcus aureus* detection (Wang et al., 2022). As shown in Figure 2C, the hydrophobic periphery prevents bacteria adhesion by maintaining a stable liquid layer. Meanwhile, the hydrophilic sensing regions capture bacteria through specific interactions between probes and target bacteria. PLIS reduced bacterial loss in the non-detection region, enabling the ultrasensitive surface-enhanced Raman scattering (SERS) detection of *Staphylococcus aureus* with the detection limits of 2.6 CFU/ml.

In addition to the anti-adhesion, the liquid-repellency of the LIS surface has also been utilized to enhance biosensing. Taking advantage of the weakly interacting interface, the three-phase contact line of droplet/air/liquid-infused surface is almost pining-free. Yang et al. developed a perfluorinated liquid-infused slippery surface platform to control the evaporation of droplets for SERS signal enhancement (Yang et al., 2016). During the evaporation process, the droplet contact angle remains constant, while the contact area decreases gradually. Therefore, as shown in Figure 2D, LIS contributes to the enrichment of analyte molecules in a small area after droplet evaporation, thus realizing ultrasensitive SERS detection. Notably, benefiting from the omniphobicity of perfluorinated liquid, the analytes in low surface energy droplets can also be enriched on LIS. According to this strategy, LIS was also employed in the signal amplification process of aggregation-induced emission (AIE) probes (Wu et al., 2021). Furthermore, LIS can be integrated into a microfluidic system for near-lossless liquid manipulation and complex assays (Wang et al., 2018; Li et al., 2020).

The sliding behavior of droplets on LIS is very sensitive to the composition of the droplets (Wang et al., 2019; Gao et al., 2020). Gao et al. demonstrated that the sliding speed of droplets on organogel infused by n-decane can be regulated by changing the chain length of single-stranded DNA (ssDNA) within the droplets (Gao et al., 2018). This work adopted ATP-induced roll-circle amplification (RCA) to generate long ssDNA. For droplets without ATP, the short ssDNA in the droplet act as the hydrotrope to increase the hydrophobic interactions between droplet and organic liquid, thus restricting the droplet sliding (Figure 2E). On the contrary, long ssDNA produced by ATP-induced RCA does not affect the sliding of droplets owing to the reduction of exposed hydrophobic groups (Figure 2E). Based on this principle, by regulating the amount of ssDNA, the sliding behavior of the droplets has been used as the output signal for detecting ATP, miRNA, and thrombin.

According to the above discussion, the introduction of LIS facilitates the development of biosensors by leaps and bounds. The anti-bioadhesion and liquid-repellency properties of LIS offer more possibilities for detection in a wide range of biological samples. With continuous efforts in detection technology, LIS is expected to be widely used as an emerging biosensing interface to improve biosensing performance.

## 5 Conclusion and prospects

LIS has attracted increasing attention from the academy and industry as a liquid interface. In this mini-review, we have summarized the recent progress of LIS in biomedical-related fields, including bioengineering, biomedical devices, and biosensing. As the novel coating, numerous spin-off companies aim to translate the LIS technology into a wide range of medical products. Despite the impressive progress, LIS is still in its infancy, and some critical challenges and issues still need to be resolved.

1) The understanding of the dynamic wetting process of droplets on LIS at the molecular level is vague. The anti-adhesion process of protein, cells, and bacteria should be revealed at the microscopic scale. A comprehensive interaction mechanism is helpful in material design and will pave the way for future applications (Daniel et al., 2017).
2) The durability of the infused liquid is another challenge for LIS toward practical application. All the properties of LIS rely on the stability of the liquid layer. The loss of infused liquid through various routes is inevitable. Therefore, LIS with long-term stability under biomedical conditions is an essential direction for future research (Baumli et al., 2021).
3) Biosecurity and biocompatibility of infused liquid should be explored intensively. Although the commonly used infused liquid such as silicone oils and fluorocarbons are considered non-cytotoxic, the effects of LIS on cell metabolism, cell differentiation, and the human immune system should be thoroughly evaluated in the long-term practice (Mackie et al., 2019).
4) Finally, with the development of infused liquid, LIS with multiple functions/properties, such as stimuli-responsive liquid-infused surface and liquid crystal-infused surface, have attracted great interest. More attention should be paid to these LIS to enable the more complicated tasks in biomedical and biosensing applications (Lou et al., 2020; Xu et al., 2021).

In brief, there are still some shortcomings of LIS in practical applications. We hope this mini-review will contribute to the advancement of novel materials and sensing interfaces in medical fields.
